# The Gas Fire Temperature Measurement for Detection of an Object’s Presence on Top of the Burner

**DOI:** 10.3390/s20072139

**Published:** 2020-04-10

**Authors:** Andrzej Milecki, Dominik Rybarczyk

**Affiliations:** Division of Mechatronics Devices, Poznan University of Technology, M. Skłodowska-Curie Square 5, 60-965 Poznan, Poland; dominik.rybarczyk@put.poznan.pl

**Keywords:** gas burner, temperature, thermocouple, IR diode, pot recognition

## Abstract

This article covers the topic of temperature measurement on top of a gas burner fire in order to recognize pot removal from a gas burner and subsequently, to cut off the gas supply. The possibility of applying a factory-mounted thermocouple was investigated with the assumption that its output signal could be used to detect the presence of a pot on a gas burner. However, the characteristic of such a thermocouple is not fully linear and as the research has shown that such a thermocouple would not fit enough for the assumed purpose, thus another sensor needs to be used. Therefore, in this paper, the linear thermocouple and IR diode are used. The best localizations of theses sensors were investigated in order to obtain a signal suitable for the pot presence recognition over the burner. These investigations are supported by the use of an infrared camera. In the investigations, the temperature changes also caused by casual air blast or caused by increasing and decreasing the valve opening are recorded and analyzed. Finally, the changes of the thermocouple’s signals are used as an input signal to propose an algorithm for pot absence recognition over the burner. The microprocessor-based circuit with a control unit for detection of the pot absence is designed, built and investigated.

## 1. Introduction

Different methods have been developed for the measuring of different flame temperatures [1,2]. These methods can be divided into probe thermometry (thermocouple- or thermoresistor-based), optical thermometry, flame gases density based and laser-based measurements. The first method is most frequently applied because it is inexpensive and easy to use. An important problem of thermocouple application is its small output voltage signal. During the burning process, a large amount of energy is released and distributed unevenly. In the flame there are also radiation losses, temperature field distortions and thermocouple inertia [3,4]. Additional sources of measurement accuracy are present in the flame: soot, liquid drops, solid crystals particles etc. [5]. Nevertheless, the application of thermocouple in gas flame temperature measurements has several advantages over other methods, like: low cost, high measurement accuracy (error in the range of 1–5%), very small thermocouple dimensions, high resistance on temperatures and gases. One of the fundamental research problems in the field of gas application is the safety of the used devices. The applications that use flammable liquids or gases are safety critical, but in some cases, the role of safety is underestimated. An example is gas cookers, in which the danger of leaving burning gas without a pot is not addressed. In paper [6], the Functional Failure Identification and Propagation analysis framework is proposed to assess the safety risks. The problem of detecting defects and emergency states is very important and therefore it has been undertaken in many works. An example is shown in paper [7], in which possibilities of defects being found in wire ropes using dynamic analysis are described. Natural gas or LPG are still widely used in households, mostly because home gas cookers are cheaper compared to electric heaters. Gas is easy to use and generates a lot of heat almost immediately after lighting the cooker. Moreover, natural gas is regarded as a clean fuel. However, gas can be very dangerous, because it is flammable. Therefore, preventing its outflow, the detection of its volatilization and the control of its consumption, are crucial. The main issue is to assure the proper use of gas in everyday exploitation, because not only can it be a source of losses, but even lead to domestic fire incidents. National Fire Incident Reporting System in US shows that cooking-related fires are the leading cause of building fires. Therefore, it is very important to cut off the gas supply after the pot is taken from the burner. In every gas home cooker produced nowadays, a thermocouple is used as the safety switch element, which cuts off the valve when there is no flame in the burner. Sometimes it happens that when using a gas cooker, the user forgets to turn it off and the gas keeps on burning. It would then be easier to have a sensor and controller to shut the gas supply when detecting that the pot was removed. Some producers use additional switch or infrared sensors to detect the presence of the pot and to switch off the gas valve. However, such solutions are cumbersome in practice because these sensors are easily soiled and their effectiveness deteriorates; their application does not guarantee the required reliability. Therefore, in order to propose a more convenient and more reliable solution to improve pot detection on a burner, research on the possibility of using a thermocouple or IR diode output signal has been undertaken in this paper. In this paper, the results of the gas flame temperature measurement are presented when the pot was put on and removed from the gas burner. Such investigations based on the system for pot recognition on the gas burner are not presented in the literature. The investigations results showed the best location of the thermocouple and IR diode for the temperature changes measurement, which may be used for recognition of the pot’s presence or not, on the gas burner. The made investigations are original and are a contribution to knowing the difference in gas flame temperature distribution when the pot is on the burner and when it is not there. So far, the presented solution in this paper has not yet been offered by any gas cooker manufacturer. 

## 2. The Safety Problem in Gas Cookers

In the cooking industry, in order to ensure that flammable gas would not flow unignited (which could lead to explosion), a special useful electro-valve is used on every pipe. The valve is opened manually by the user, but its opening is sustained by electricity generated by the thermocouple located in a flame. So, the electro-valve is controlled through the special thermocouple installed below every burner. If there is no flame on a burner, the energy generated by the thermocouple is too small to sustain the valve opening and as a result the valve switches off the gas supply. In this way, every burner is protected against gas leakage. However, these types of thermocouples are mass-produced most often by companies in China that do not publish the exact characteristics of these thermocouples.

**Figure 1:**
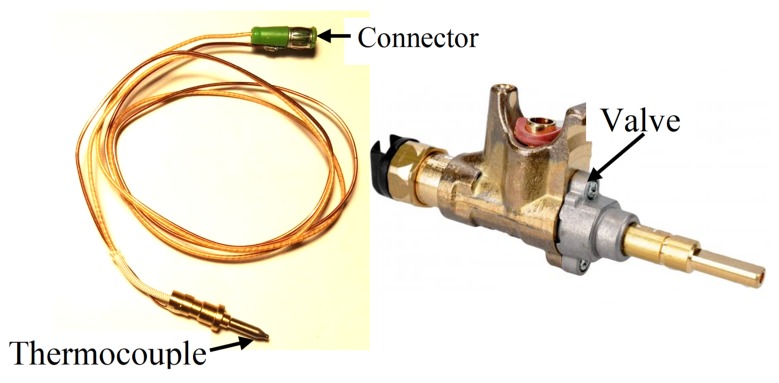
The gas electromagnet valve with the thermocouple [6].

In Figure 1, the example of the gas magnet valve and the thermocouple, which can provide the electric output power, is shown [8]. Such thermocouples and valves are widely used in many gas appliances, like gas cookers, gas stoves, gas ovens, gas heaters and so on. They include a flameout safety protector, which can be delivered in different styles and sizes. When the thermocouple tip is heated by the fire, it generates the electric power. This electric power actuates the magnetic valve, which remains open to the gas flow. When the flame is out, the electric power generated by the thermocouple decreases rapidly and as a result, the valve is closed through the action of its spring. Typically, it is 600–700 °C on the flame. At such a temperature, the thermocouple output electric potential is higher than 15–25 mV, which is sufficient to supply the electro-valve. When there is no flame, the thermocouple output voltage is below 1.5 mV.

In paper [9], the mechanisms for ignition and extinction for condensed-phase fuels via the use of a gas-fuelled burner are explored. The investigation goal was to specify a set of mass fluxes of fuel mixture that corresponded to the flash-fire and extinction points. The results showed an increase in critical mass flux with a decreased heat of combustion. An anchor point is proposed to establish when quasi-steady burning is initiated. The papers [10,11] propose a methodology for assessing the frequency of ignition and exposure of consumer products in a building, in which an auxiliary heating device (electric heaters, wood stoves) is the ignition source. The article [12] presents a building fire risk analysis model based on scenario clusters and its application in fire risk management of buildings. The average fire risk of residential buildings in China is quantified. 

There are several patents related to the use (and its extension) of the thermocouples in gas cookers. For example, patent [13] proposes using the thermocouple to find the fire in the apartment. This invention relates to fire detectors, and more particularly, to fire detecting devices utilizing a series of thermocouple elements as the detector and signal initiating device. In patent [14], the authors have described the dedicated construction of a gas cooker with thermocouples and special designed control system, which uses multiple thermocouples in series to permit the safe operation of the gas burner. The control system produces signals to regulate the flow of fuel to the gas burner. The thermocouples for measuring the flame temperature, the combusted gas temperature and the overall chamber internal temperature were used in the controller.

In patent [15], another invention is proposed, which relates to the operation of unvented gas fired appliances. It enhances the safety of effectively controlling the flow of gases to a gas heater. In the solution, the thermostat controls the temperature of the heated area by combining a thermocouple for safety and a thermopile for operation of the thermostat. The thermocouple shuts off the gas when the pilot flame goes out, and the thermopile operates the thermostat to have a desired room temperature provided by the gas fired burner.

In paper [16], a correctional calculation method is proposed for measurements of temperature profiles in flames with a thermocouple. This method includes an iterative procedure of correcting a temperature profile with respect to heat losses. Experiments were performed on town gas–air flames. The accuracy of this correctional method is discussed by taking into consideration the influence of several parameters on heat transfer. Three correction and extrapolation methods of temperature measurement by thermocouple were developed in [17]. A systematic evaluation of validity for these methods was proposed. Analysis has shown that the flame turbulence imposed a negative effect on the correction methods. However, the extrapolation method could correct the fire gas temperature regardless of temperature pulsation. The thermocouples may be used for early fire detection in buildings, measuring the temperature gradient close to the ceiling in the room, caused by ascending smoke plumes [18]. The investigation’s results have shown that thermocouple measurement is accurate in such cases and the authors have proposed the application of thermocouple measurement for fire detection in buildings. In paper [19], two methods of measuring the smoke layer are used: ocular estimate and thermocouple measurement, showing that these two methods can be complementary.

**Figure 2:**
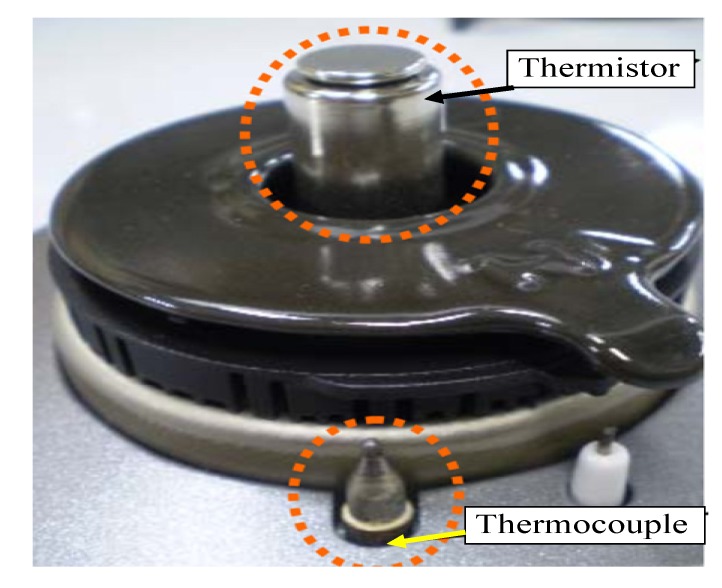
The photo of the SI sensors [22].

As reported in [20,21], in Japan, about 4507 house fires were caused by gas stoves in 2007. To decrease this number of house fires, it has been required in Japan that all gas stoves should be equipped with a safety system, called “Si” sensors, made up of a thermocouple and a thermistor. The “Si” sensor is an acronym that is composed of the initial letters of words: Safety, Support, Smile and Intelligent. The first one is used for flames detection, whereas the second one measures the temperature of the pot on the burner. The thermistor serves as an automatic switch which turns off the gas when users forget to do so. The paper [22] describes the development of gas stoves with “Si” safety sensors. In Figure 2, the photograph of the “Si” sensor is shown. The sensor switches off the valve when the temperature of the live flame in the burner is above 250 °C. Unfortunately, the disadvantage of the proposed sensor is its troublesome cleaning. In the paper quoted here, the new trials to create extended functions and products for the future are also described. Several ongoing research and development projects are shortly described, like the development of a new gas stove named “Gas Pad” performed by Tokyo Gas Co. In the designed stove, several distinguishing characteristics, including a thin, lightweight body and control system, were implemented.

## 3. The Application of the Thermocouple in Gas Cookers

**Figure 3:**
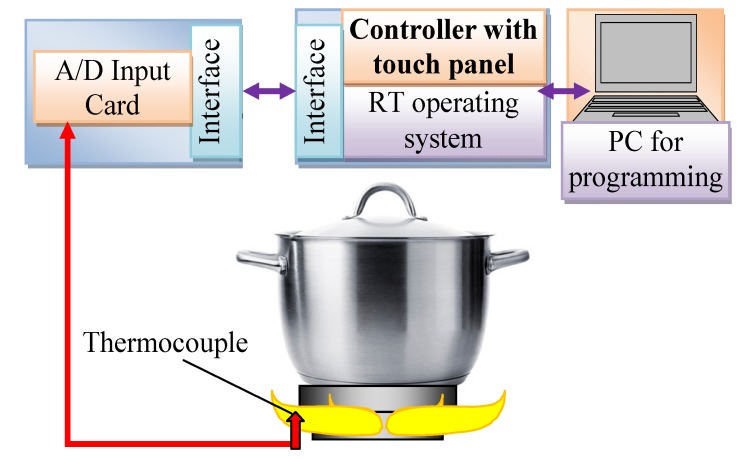
Scheme of the test stand.

**Figure 4:**
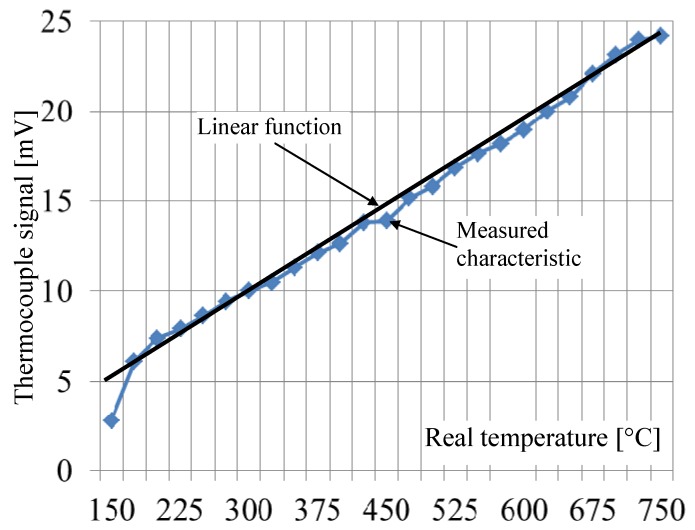
Factory mounted thermocouple characteristics.

As mentioned above, in every gas oven, a thermocouple is used. In the first attempt of the described tests here, this sensor is applied for the recognition of pot presence on the burner, and it was considered whether its signal could be used successfully for this purpose. In Figure 3, the scheme of the experimental stand for testing of the factory mounted thermocouple (FMT) in a cooker is shown. Amica (Hansa, Gram, CDA) company gas plate type GC0620 was used in the tests. In the tests, the medium burner with a heat load of 1.75 kW and energy efficiency (EE gas burner) 54.5, was used. As a fuel, propane butane gas (3B/P G30/37mbar) was used. This stand is based on a controller working under a real-time operating system. This controller is connected by a serial interface with analog/digital card with special thermocouple inputs, to which a thermocouple was connected. The measured data are transmitted to the PC, which stored it on an HDD. The PC was additionally used for the programming of the controller. The complete system enabled the real-time measurement of the thermocouple output signal with a sampling frequency of 1250 Hz. The measured static characteristic of the factory mounted thermocouple is shown in Figure 4. It is available as a spare part under the name Amica Somipress S1. The thermocouple tip is placed 3.8 mm from the flame nozzle outlet. It is visible in this figure that this characteristic is almost linear for temperatures above 200 °C. The output voltages are in the range of 6 mV to 24 mV for temperatures from 150 °C to 750 °C, which means that the factory mounted thermocouple resolution is 0.032 mV/°C. 

**Figure 5:**
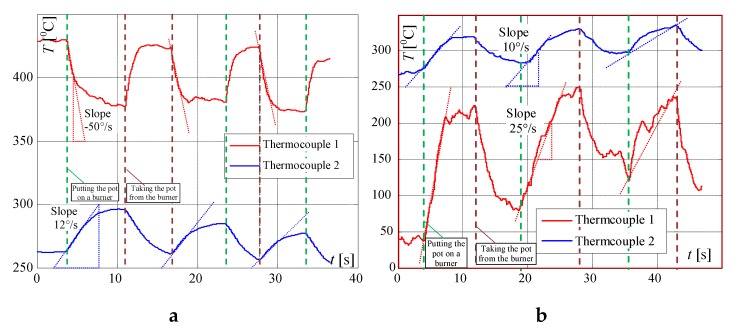
Temperature changes measured by K-type thermocouples while operating the valve (opening and closing): (**a**) without a pot; (**b**) with a pot.

**Figure 6:**
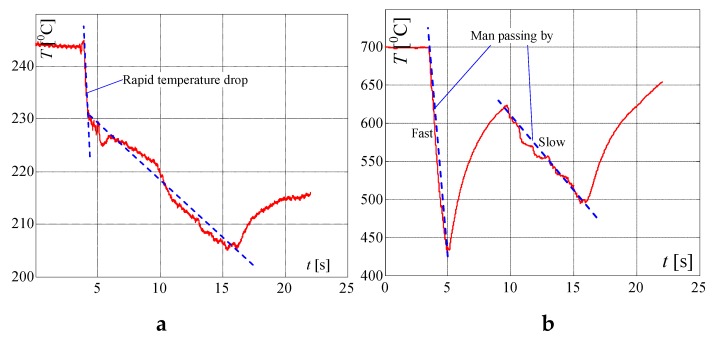
Temperature changes during non-controlled air blow caused by: (**a**) opening the window when the flame is small; (**b**) quick and slow human movement when the flame is big.

In the next investigations, the changes in signal obtained from two K-type thermocouples located in two different positions were recorded while the valve opening was changed. The curves were recorded when the pot was on and off the burner and set in Figure 5. These investigations have shown that if the pot is present on the burner, the flame temperature may increase or decrease, depending on the thermocouple location. In Figure 6, the results of the influence on the air blow on the flame temperature measurement are investigated. It is visible that the air blow caused the human to walk parallel to the front of the oven at a distance of about 0.5 m and resulted in a rapid and significant change (decrease) in the measured temperatures. In these cases, the shape of the temperature change curves can be very diverse (accidental) and the temperature signal slope is very high in comparison to the temperature changes slope caused by the operation of the gas valve. These examples give an insight into the problem of eliminating interference in the process of recognizing the presence of a pot.

**Figure 7:**
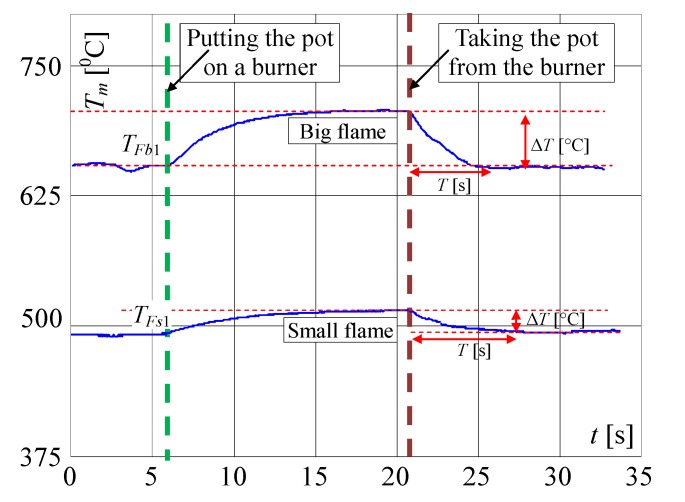
Temperature changes measured by a factory mounted thermocouple when putting the pot on and removing it from the burner, when the flame is big (valve on max.) and small (valve on min.).

In the next investigations, the temperature changes for different heights of flame are measured when the pot is placed on and removed from the burner. In this article, only the results for maximum and for minimum valve openings are included. The term "big flame" will be used when the burner gas supply valve was fully open. The term “small flame” will be used to describe the minimum flame retention in the cooker. It is worth noting that the slopes of the recorded curves are significantly smaller than the signal’s slopes presented in Figure 5 and Figure 6. This feature can be used to distinguish whether the change in measured temperature was caused by taking or placing the pot on the burner, or by other reasons like air blow or change of the valve opening. In Figure 7, the signals recorded by the factory mounted thermocouple are shown. 

**Figure 8:**
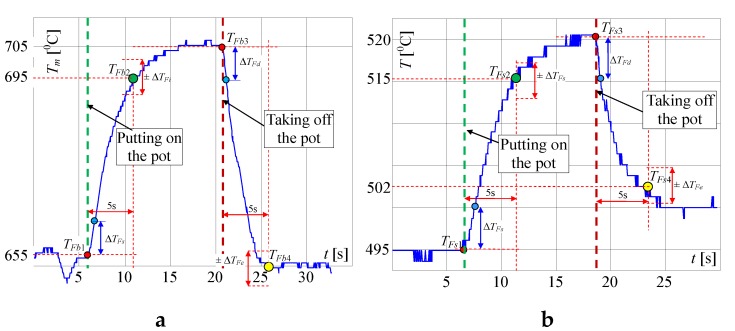
Extended characteristics measured by a factory mounted thermocouple if putting the pot on and removing it from the burner, when the flame is: (**a**) big; (**b**) small; explanation: red circle—start points of temperature changes: *T_Fb_*_1_, *T_Fb_*_3_ and *T_Fs_*_1_, *T_Fs_*_3_; blue circle—points for recognition that the temperature changes significantly within 1s; green circle – recognition that the pot is placed on the burner *T_Fb_*_2_, *T_Fs2_* (temperatures after 5 s); yellow circle—recognition that the pot is taken from the burner *T_Fb_*_4_ and *T_Fs_*_4._

In Figure 8, the same but enlarged curves are presented. When the flame is high (valve open on maximum) and the pot is not on the burner, the measured temperature is *T_Fb_*_1_ = 655 °C (Figure 8a). If the pot is placed on the burner, the temperature increased to about 695 °C in the time *T* = 5 s (Δ*T* = 40 °C) and within the next 5 s to 705 °C. To achieve the goal of the research, the measurement of the temperature change when the pot is taken away from the burner is the most important. In this case, as registered, the temperature decreased in the time of *T* = 5 s from 705 to 655 °C i.e., Δ*T* = 50 °C. In the case of a small flame (Figure 7) i.e., when the valve is open on minimum, the temperature increased in 5s from 495 to 515 °C i.e., only of Δ*T* = 20 °C and decreased from 520 to 502 °C i.e., only of Δ*T* = 18 °C, also in *T* = 5 s. Such rather small temperature changes may be too difficult to recognize automatically that the pot is absent, using only a factory-mounted thermocouple. 

**Figure 9:**
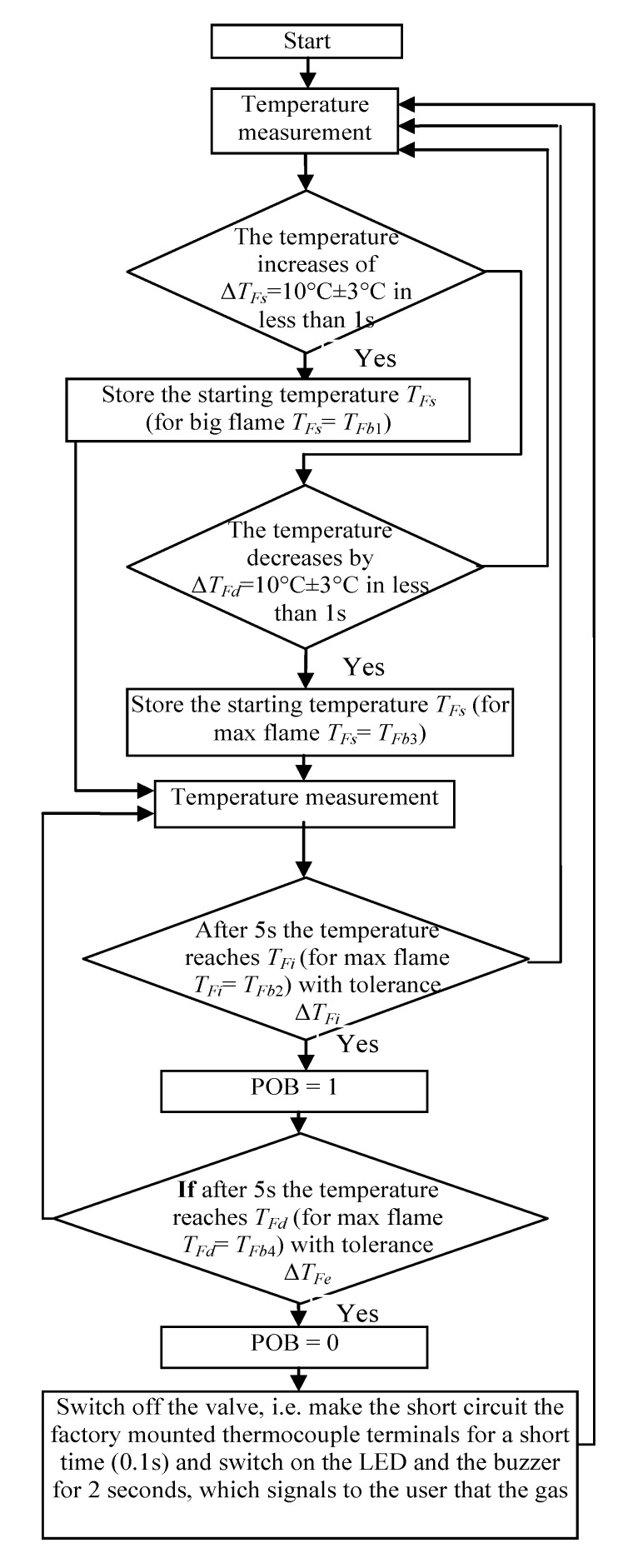
Temperature changes measured by linear thermocouple located in position 3.

In the first attempt, the above described signals for the big flame were used for checking whether it is possible to detect the taking of a pot from the burner, using only a factory-mounted thermocouple. In Figure 8, on the presented curves the small red, blue, green and yellow circles are plotted, which show the characteristic points detected and used by the proposed algorithm. At first, the significant changes of the temperature were detected, using points (circles) denoted on curves: red point, which shows the start of the temperature change, and blue point, which shows that this change is significant. For the big flame, the following simplified steps were implemented in the Algorithm 1 for recognition that the pot is taken from the burner (Figure 9):
**Algorithm 1**1: **If** the temperature increases of Δ*T_Fs_* = 10 °C ± 3 °C in less than 1 s (changes from red circle to the blue one), **Then** store the starting temperature *T_Fs_* (for big flame *T_Fs_* = *T_Fb1_*) and **go to** point 3.2: **If** the temperature decreases by Δ*T_Fd_* = 10 °C ± 3 °C in less than 1s (changes from red circle to the blue one), **Then** store the starting temperature *T_Fs_* (for max. flame *T_Fs_* = *T_Fb3_*) **go to** point 5, **Else** go to point 1.3: **If** after 5 s the temperature reaches *T_Fi_* (for max. flame *T_Fi_* = *T_Fb2_*) with tolerance Δ*T_Fi_* (see Figure 8), **Then** set the indicator “Pot on the Burner” (PoB) to 1, **Else go to** point 1.4: **If** after 5 s the temperature reaches *T_Fd_* (for max. flame *T_Fd_* = *T_Fb4_*) with tolerance Δ*T_Fe_* (see Figure 8), **Then** set the indicator POB to 0 (“pot taken from the burner”), **Else go to** point 3.5: **If** POB is equal to 0, **Then** switch off the valve, i.e., make the short circuit the factory mounted thermocouple terminals for a short time (0.1 s) and switch on the LED and the buzzer for 2 s, which signals to the user that the gas flow is not turned off after removing the pot.6: **Go to** point 1.


Because the algorithm should work properly for every flame between small and big, mentioned in the algorithm the temperatures *T_Fi_* (green circle) and *T_Fd_* (yellow circle) must be calculated using temperatures measured for min. and max. flame, presented in Figure 8. To these calculations, two linearizing equations were used. When the temperature increased, the following equation was used in the made investigations:

(1) $$T_{Fi}=\frac{695-515}{655-495}\left(T_{Fs}-495\right)+515=3\left(T_{Fs}-495\right)+515$$

where: *T_Fb_*_2_ = 695 °C and *T_Fs_*_2_ = 515 °C; *T_Fb_*_1_ = 655 °C and *T_Fs_*_1_ = 495 °C—temperatures taken from Figure 8a,b for max. and min. flame; *T_Fs_* is starting temperature recorded by the algorithm in point 1 or 2. 

When the temperature decreased following equation was used in the algorithm for calculation of temperature *T_Fd_* used in point 4:

(2) $$T_{Fd}=\frac{650-502}{705-520}\left(T_{Fs}-520\right)+502=0.26\left(T_{Fs}-520\right)+502$$

where *T_Fb_*_4_ = 650 °C, *T_Fb_*_3_ = 705 °C is a big i.e., max. flame (see Figure 8a); *T_Fs_*_4_ = 502 °C, *T_Fs_*_3_ = 520 °C—temperatures taken from Figure 8a,b for max. and min. flame respectively.

These equations were formulated based on the experimental investigation results (signals) obtained from factory mounted thermocouple, not included in this paper. The experimental tests showed that the temperatures *T_Fi_* and *T_Fd_* can vary from those calculated using Equations (1) and (2) by approx. 10%, therefore such tolerances are added in point 2 of the algorithm. These tolerances are calculated using following equations:

(3) $$T_{Fi}=T_{Fi}\pm 0.1\cdot \left(T_{Fi}-T_{Fs}\right)$$

(4) $$T_{Fd}=T_{Fd}\pm 0.1\cdot \left(T_{Fd}-T_{Fs}\right)$$

The proposed above algorithm was implemented in the controller (see Figure 3) and tested in a laboratory environment. It enabled the control of the gas flow valve in a whole range of temperatures measured by the factory mounted thermocouple. However, extensive experimental investigations have shown that the pot absence on the burner recognition for a small flame was not satisfactory in all attempts, because the influence of disturbances, like for example gusts of air caused by human movement, resulting in an unexpected reaction of the control system. Moreover, several unexpected behaviors over time were recorded. Sometimes sudden, fast drops of thermocouple voltage output signals occurred, without any changes of the flame. These drops corresponded to temperature change i.e., decrease of Δ*T* = 20 °C and were taken into account by the above described algorithm for detection of the pot removal from the burner. As a result, wrong pot presence recognition was made by the control system. It should be noted that the FMT is not envisaged for temperature measurement but only to supply the valve’s electromagnet when the gas burns. Moreover, the proposed idea to make a short circuit of this thermocouple ending may be not accepted by stove producers, because this thermocouple is only intended for safety and its use for other purposes may disturb its work. Therefore, in the next step, the linear thermocouple (LT) and additional on/off gas valve were proposed to be used, to solve the problem described above. 

**Figure 10:**
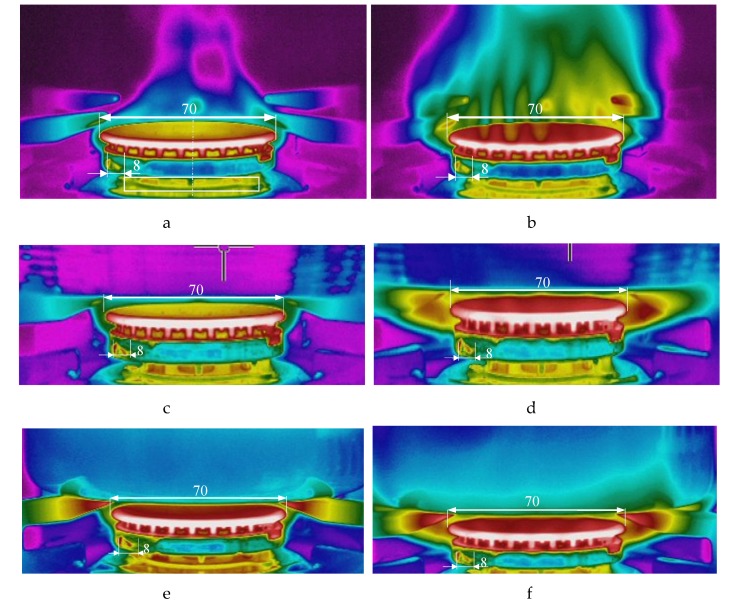
Pictures taken by the infrared camera: (**a**) small flame without a pot, (**b**) big flame without a pot, (**c**) small flame with a pot of 220 mm diameter, (**d**) big flame with a pot of 220 mm diameter, (**e**) small flame with an enameled pot of 170 mm diameter, (**f**) big flame with an enameled pot of 170 mm diameter.

Infrared thermography (passive and active) is one of the most important measurement techniques used in many areas of science and industry [23,24]. In paper [25], the detection of turbulent vortices in the flames of burning liquid hydrocarbon fuels was investigated. Theoretical predictions and experimental results of the use of infrared thermography were presented. In order to recognize the temperature distribution in the flame, the thermal imaging camera was used to measure the temperatures around the gas burner. Therefore, in the next investigations, a high resolution infrared camera type FLIR T620SC with parameters: max. temperature measurement 650 °C, accuracy ± 2%, resolution 0.05 °C, was used to find the areas of highest flame temperatures. Several photos were taken and some of them are shown in Figure 10. Even though the infrared camera can only measure the temperatures outside the flame, the hottest point of the flame can be estimated. It is visible that in every investigated case (small and big flame, without and with a pot), this point is about 8 mm from the bottom base (ring) of the burner.

**Figure 11:**
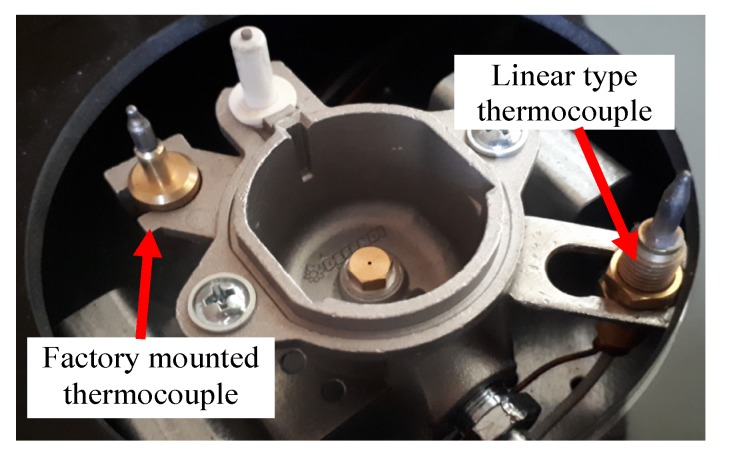
The photo of the used thermocouples.

**Figure 12:**
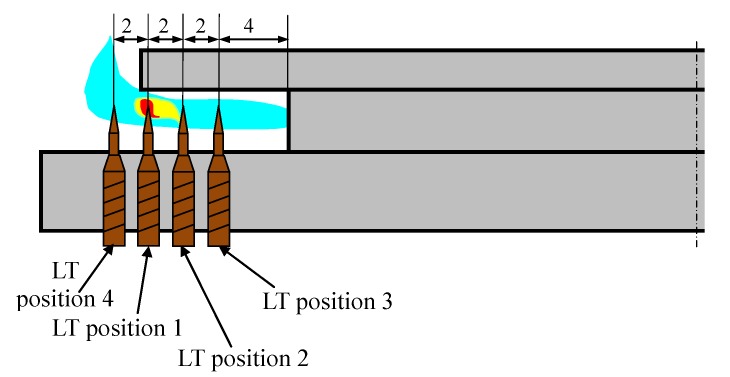
The positions of the linear thermocouple.

**Figure 13:**
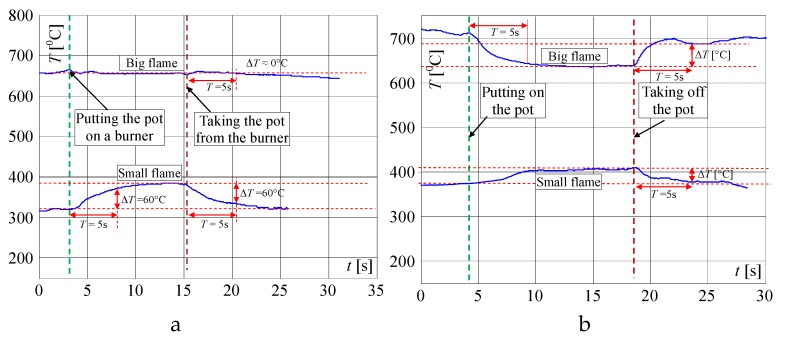
Temperature changes for putting the pot and removing it from a burner when the flame is big and small measured by linear thermocouple located in: (**a**) position 1, (**b**) position 2.

The burner in which two thermocouples are used is shown in Figure 11. At first, such a position of the linear thermocouple was sought, in which its output signal changes (temperature changes) were the greatest. Four different positions were chosen for tests, called here as: 1, 2, 3 and 4, as shown in Figure 12. The first point was taken according to the results obtained by infrared camera i.e., the LT was assembled 8mm from the bottom ring. Then, this thermocouple was assembled in the next points. The distance between these positions was equal to 2 mm. In Figure 12, the recorded temperature changes obtained from the linear thermocouple located in position 1 are shown. It should be noted that in this case, the direction of temperature changes is different in comparison to temperature change directions of the factory mounted thermocouple (compare Figure 8a and Figure 13a). In the case of a small flame, the temperature direction change was opposite i.e., the temperature increased when the pot was placed on the burner and decreased when the pot was taken from the burner. The temperature changes were also detected in the time of *T* = 5 s. Although the temperature changes were not big, it was assumed that they could be recognized and used in pot detection.

**Figure 14:**
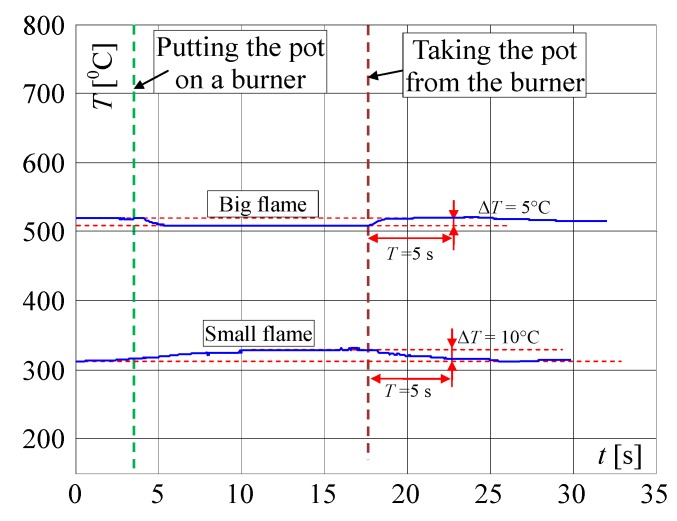
Temperature changes measured by the linear thermocouple located in position 3.

In Figure 13b and Figure 14, the same signals are presented but measured when the LT was assembled in position 2 and position 3, respectively. In both cases, the temperature reaction (changes) to putting the pot on the burner and removing it, were significantly worse than in comparison to position 1. Similarly, worse investigation results were also obtained when the LT was installed in position 4. Therefore, in the next investigations, the linear thermocouple was located in position 1.

**Figure 15:**
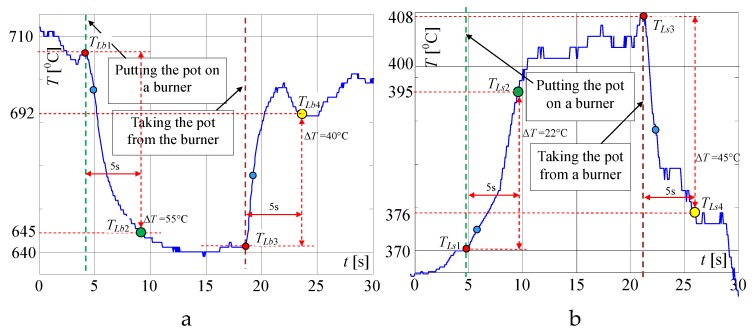
Extended characteristics measured by the linear thermocouple in position 1 (Figure 11) if putting the pot on and removing it from the burner when the flame is: (**a**) big, (**b**) small; explanation: red circles—start points for big flame *T_Lb_*_1_ and *T_Lb_*_3_ and for small flame *T_Ls_*_1_ and *T_Ls_*_3_; blue circle—recognition of temperature changes, green circle—recognition that the pot is placed on the burner *T_Lb_*_2_ and *T_Ls_*_2_; yellow circle—recognition that the pot is taken from the burner *T_Lb_*_4_ and *T_Ls_*_4._

**Figure 16:**
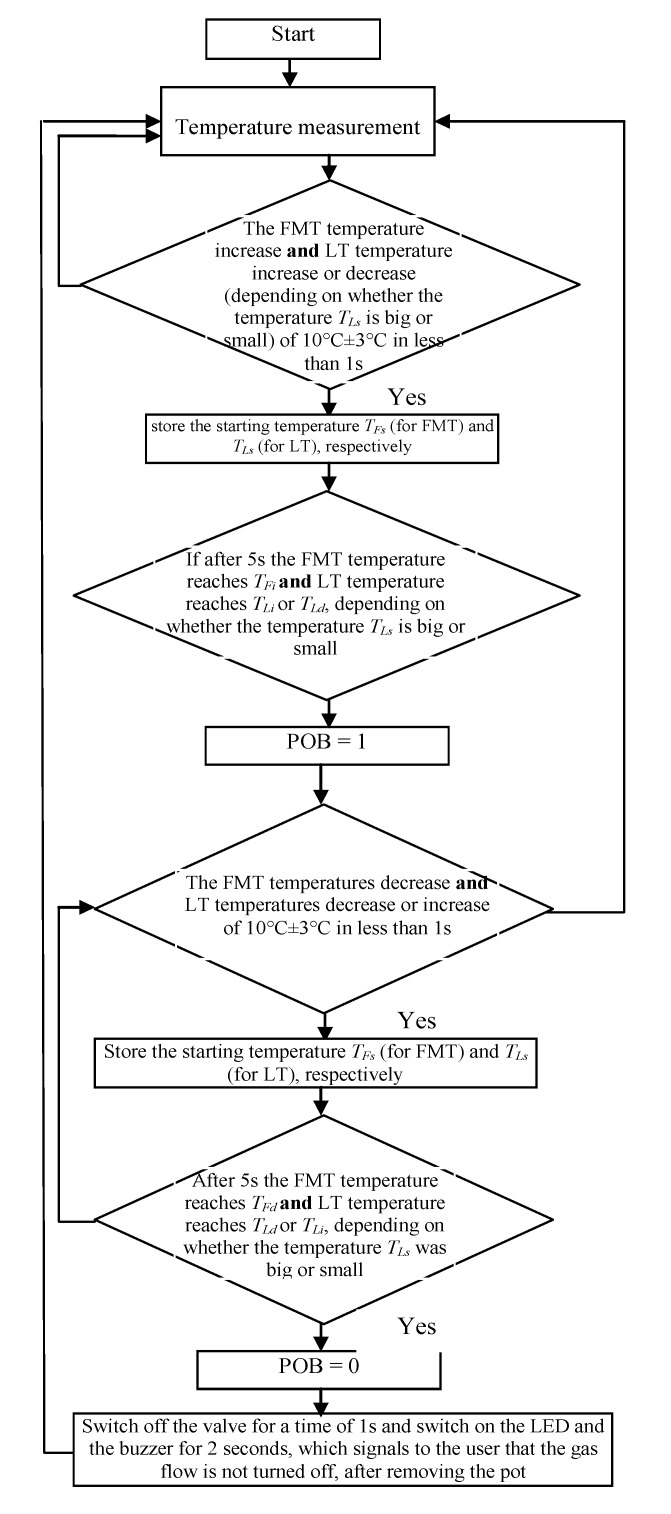
Temperature changes measured by the linear thermocouple located in position 3.

In Figure 15, the expanded signals recorded by the linear thermocouple located in position 1 (shown in Figure 13a) are presented. If the flame was big and if the pot was not on the burner (Figure 15a), the temperature changed in a range of 703 to 711 °C. If the pot was placed on the burner, the temperature decreased in 5 s from 705 to 645 °C (Δ*T* = 60 °C). If the pot was taken away from the burner, the temperature increased in the time 5 s from 642 to 692 °C (Δ*T* = 50 °C). In case when the flame was small and the pot was placed on the burner (Figure 15b), the temperature increased within 5 s from 370 to 395 °C (Δ*T* = 25 °C), and if the pot was placed on the burner the temperature decreased from 408 to 376 °C (Δ*T* = 32 °C). The temperature changes for a small flame were small, but similar to those measured by FMT. Finally, it was decided to use both thermocouples, which should significantly improve the reliability of pot detection on the burner. New rules were introduced to the above described Algorithm 2 establishing it as follows (Figure 16):
**Algorithm 2**1: **If** the FMT temperature increase **and** LT temperature increase or decrease (depending on whether the temperature *T_Ls_* is big or small, as shown in Figure 15a,b) of 10 °C ± 3 °C in less than 1 s, **Then** store the starting temperature *T_Fs_* (for FMT) and *T_Ls_* (for LT), respectively, **Else** go to point 1.2: **If** after 5 s the FMT temperature reaches *T_Fi_*
**and** LT temperature reaches *T_Li_* or *T_Ld_*, depending on whether the temperature *T_Ls_* is big or small, **Then** set the indicator “Pot on the Burner” (PoB) to 1, **Else** go to the point 1.3: **If** the FMT temperatures decrease **and** LT temperatures decrease or increase of 10 °C ± 3 °C in less than 1 s, **Then** store the starting temperature *T_Fs_* (for FMT) and *T_Ls_* (for LT), respectively, **Else** go to point 3.4: **If** after 5 s the FMT temperature reaches *T_Fd_*
**and** LT temperature reaches *T_Ld_* or *T_Li_*, depending on whether the temperature *T_Ls_* was big or small, **Then** set the indicator “Pot off the Burner” (PoB) to 0, **Else** go to the point 3.5: **If** POB is equal to 0, **Then** switch off the valve for a time of 1 s and switch on the LED and the buzzer for 2 s, which signals to the user that the gas flow is not turned off, after removing the pot.6: Go to point 1.


The temperatures *T_Li_* and *T_Ld_* are calculated using the following linearizing equations, formulated basing on recorded and presented in Figure 16 curves and parameters:

(5) $$T_{Li}=\frac{645-395}{706-370}\left(T_{Ls}-370\right)+395=0.74\left(T_{Ls}-370\right)+395$$

(6) $$T_{Ld}=\frac{692-376}{640-520}\left(T_{Ls}-408\right)+376=1.36\left(T_{Ls}-408\right)+376$$

where *T_Lb_*_2_ = 645 °C and *T_Ls_*_2_ = 395 °C; *T_Lb_*_1_ = 706 °C and *T_Ls_*_1_ = 370 °C; *T_Lb_*_4_ = 692 °C and *T_Ls_*_4_ = 376 °C; *T_Lb_*_3_ = 640 °C and *T_Ls_*_3_ = 408 °C—temperatures taken from Figure 14 for max. and min. flame.

**Figure 17:**
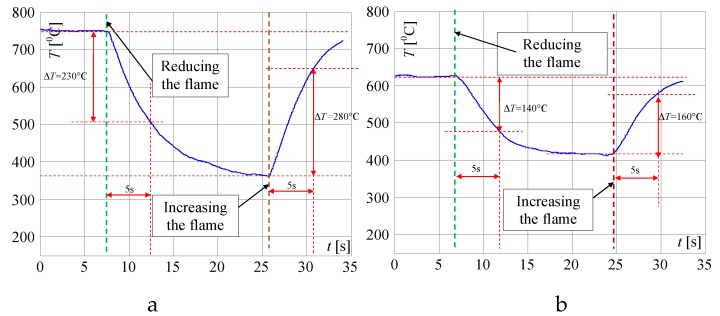
Temperature changes measured by the linear thermocouple in position 1, when the gas valve was closing and opening i.e., when reducing and increasing the gas flow to the burner if the pot is: (**a**) off, (**b**) on the burner.

In Figure 17, the signals measured when the user changed the flame are presented. In both investigated cases, the temperature changes within 5 s were significantly bigger than temperature changes caused just by putting or taking the pot on/off the burner. If the pot is removed from the burner, the recorded temperature changes within 5s are in a range of 230 to 280 °C, i.e., Δ*T* = 50 °C. If the pot is on the burner, the recorded temperature changes are in a range of 140 to 160 °C i.e., Δ*T* = 40 °C. Such temperature changes are much bigger than temperature changes recorded when the pot was put on or taken away from the burner. This feature was additionally implemented in the algorithm by adding a limit of the change range of Δ*T*. Thanks to this, the system does not react when the user changes the gas flow. The same concerns flame changes during non-controlled air blow caused by opening the window when the flame is small or human movement near the gas cooker (Figure 6). 

## 4. The Application of the Infrared (IR) Diode

In paper [23], the thermocouple investigation results are presented. These tests are made in order to measure its sensitivity to fluctuations in flames and to determine if the average thermocouple output signal was representative of the local volume temperature for fluctuating flames. The experiments have illustrated the complexity of the nature of the measurement temperature in flames using thermocouples. The article confirmed that if average temperature is the quantity sought, as it is in described in this paper’s case, then thermocouple measurement alone is not sufficient to provide complete information. Similar statements can be found in many references describing the problems of thermocouple measurements of fluctuating flame. Therefore, it is worth considering using another sensor. 

**Figure 18:**
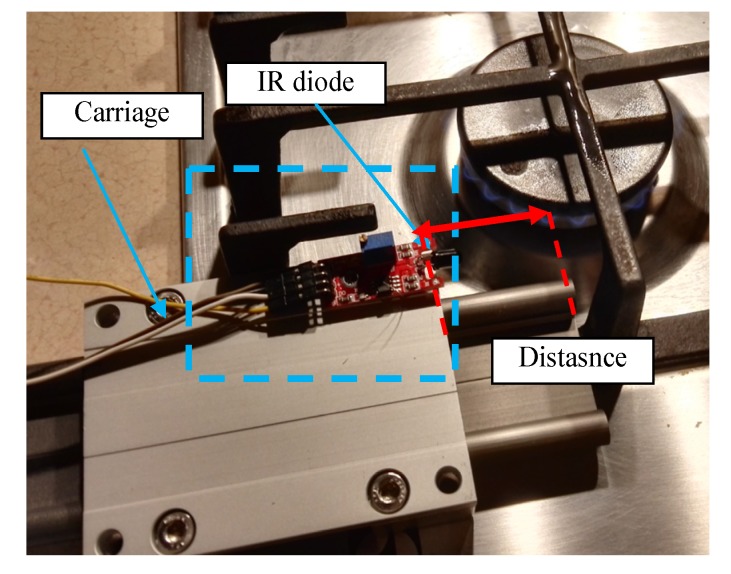
The application of the IR diode for the temperature measurement.

**Figure 19:**
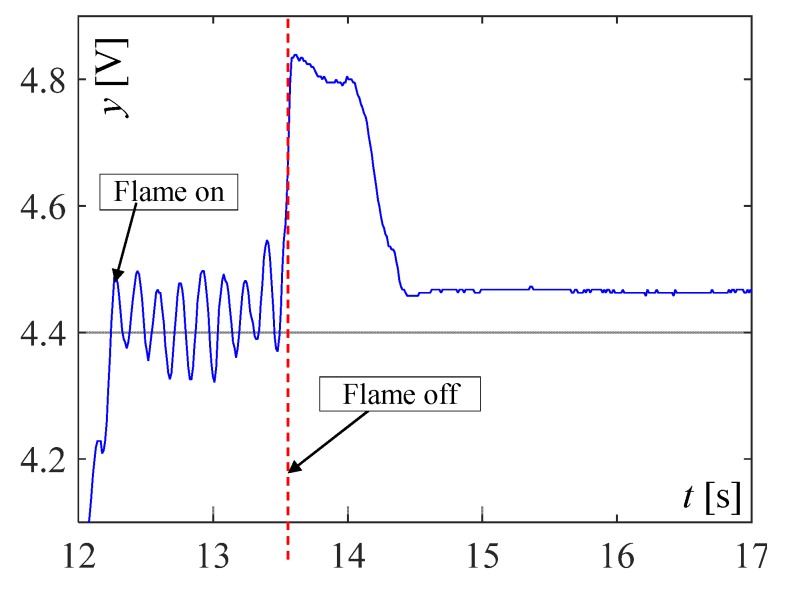
IR diode output signal if the flame is on and off.

The visible and infrared radiation is 99% of the whole spectrum of gas flame radiation, therefore, in the next investigations, low cost IR photodiode type SFH203 with additional electronics type LM393 (voltage comparator), was also used (Figure 18). Its output signal was a voltage changing in a range of 0 to 5 V. In fact, in the flame measurement only the highest values of output signals are used i.e., above 4.3 V. The IR photodiode measuring range of wave’s length is from 760 to 1100 nm, which is suitable for gas flame temperature measurement. This is because the flame highest radiation is above the wave length of 900 nm. In Figure 19 the recorded temperature curves are shown; it can be seen that if the flame is on, the signal is oscillating. These oscillations are caused by flame flickering. In the absence of a flame, the measured signal was not oscillating. Moreover, the amplitude of the signal oscillation changes i.e., decreases in the presence of a pot and increases in the absence of the pot. In Table 1, the recorded temperature changes measured by IR photodiode, which was located at a distance of about 50 and 80 mm from the flame, are presented. The same investigations are made when the IR photodiode was located about 110 mm from the flame. In the obtained signals, the oscillation amplitudes are measured. For every case, the differences between amplitudes occurring when the pot is present above the flame and when it is not present are calculated. The results i.e., these differences between the signal amplitudes, are summarized in Table 2. Additionally, in this table the amplitudes of measured signals by the IR photodiode, located about 110 mm from the flame, are included.

There biggest difference between the values of the signal’s amplitude for the presence and absence of the pot was obtained when the diode was close to the flame, i.e., 50 mm. The smallest change in oscillation was visible when the distance from the flame was 110 mm. Nevertheless, even this change can be easy detected by the algorithm implemented in the controller. 

The advantage of using an IR diode compared to a thermocouple is the much better time constant and no memory effect. Moreover, the cost of such diodes with dedicated electronics is low, which is very important for gas cooker producers. The disadvantage is the need to install an additional element in the gas stove, which must also be protected against direct flames and must be periodically cleaned.

## 5. The Design and Investigation of the Controller

**Figure 20:**
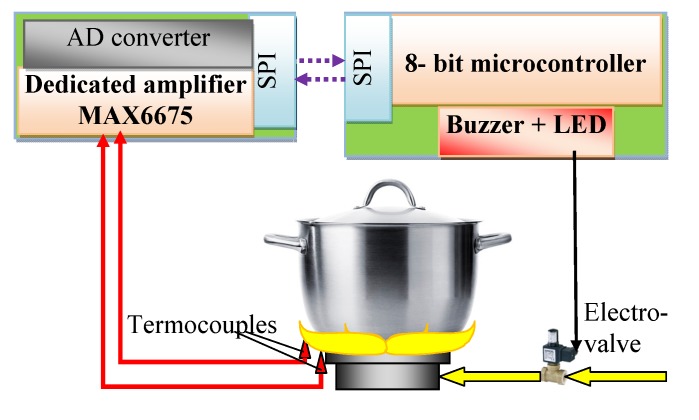
Scheme of the electronic controller.

**Figure 21:**
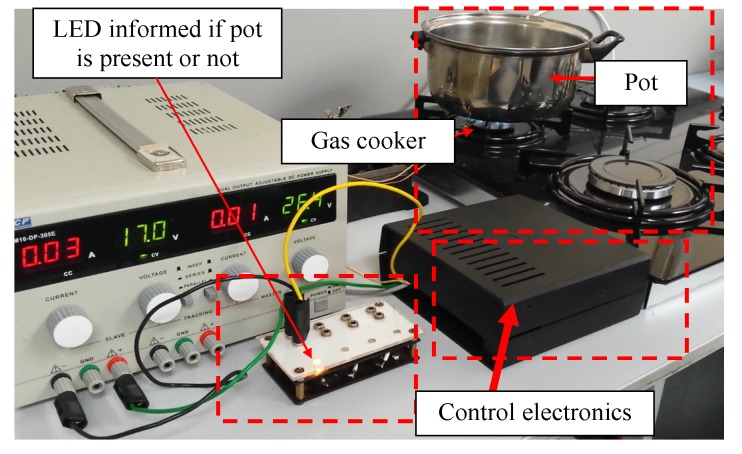
Photo of the test stand with the electronics.

The microprocessor-based device that is able to process temperature signals measured by the thermocouples and to control i.e., switch on and off the gas valve, was built by the authors. LED and a piezoelectric buzzer were embedded in order to signalize pot removal. In this device, the above described algorithm was implemented and tested. The changes of the thermocouple signals were used as the input signals in this electronic control system. Its main task was to detect the pot presence and absence on the gas burner and to cut off the gas supply if the pot is taken by the user from the burner. In Figure 20, the structure of the built controller is shown. In Figure 21, the photo of the test stand on which the built controller was used is presented. 

**Figure 22:**
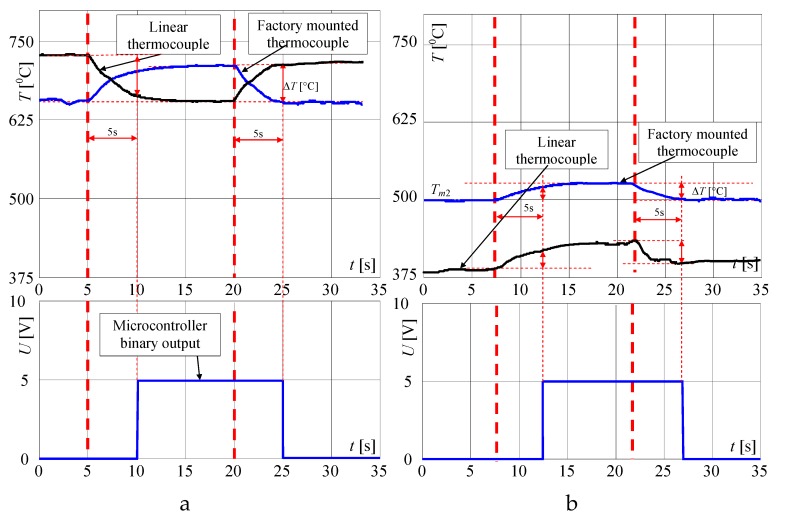
Signal changes measured by both thermocouples for the placing and removing of the pot from a burner if the flame is: (**a**) big, (**b**) small, and microcontroller binary output signal.

In Figure 22, the temperature signal changes measured by both used thermocouples during the placing/removing of the pot in the case of a big flame are summarized. The algorithm recognized the point in which the signal from the factory mounted thermocouple decreased and the signal from the linear thermocouple increased. In the next step, the algorithm sampled the signals for about 5 s, and if in this time the temperature measured by both thermocouples decreased or increased respectively by more than about 20 °C, the microcontroller generated the sound signal, switched on the LED and after the next 5 s, switched off the valve. 

The investigations confirmed that the developed solution worked well for a high flame, when both signals from thermocouples are used, and when only one signal from i.e., a linear thermocouple, is used. In the performed investigations, another algorithm, in which the decision of if the pot is present on the burner or not was made after 3 s or even after 2 s after observing the change in temperature. The obtained results were also quite satisfactory in such a case.

However, the investigation results have shown that for a small flame, the recognizing of the removal of the pot from the burner was difficult due to a small temperature change and sometimes the pot removal was not detected. That is why it was decided to switch off the valve i.e., cut off the valve only for a big flame. For small flames, the system only switched on the LED and the buzzer for 1 s, giving the user information that probably the gas is burning without the pot.

The above described solution may find application in modern gas kitchens in order to turn off the gas supply when there is no pot over the flame. The system will generate a warning sound, light (flashing LED) and will shut the gas valve off, in the absence of the pot on the burner.

## 6. Conclusions

The main purpose of the work presented in this paper is to design and build a microprocessor-based unit able to cut off the gas flow to the burner when the pot is removed. At first, the results of the investigations of a factory mounted thermocouple are described. This thermocouple is commonly used as a safety element tasked to switch off the gas flow through the valve when there is no flame in the burner, i.e., when the gas reaching the burner does not burn. The possibility of its use to detect the presence of a pot above the burner was considered and investigated. Such a solution would be most suitable for cooker producers, because it could be implemented without any significant changes in the cooker’s design. However, the tests have shown that the use of the signal generated by the factory-mounted thermocouple may result in unacceptable number of errors. This thermocouple may exhibit unexpected behaviors, i.e., change of the output signal in dynamic states. Moreover, extending its usage outside what it was designed for could adversely impact the basic application. Therefore, a second (additional) linear thermocouple was assembled and used. The characteristic of this thermocouple was linear. At first its, best mounting position was sought and confirmed. In this position, the signal changes enabled the recognition of the moment in which the pot was placed on the burner and the moment in which it was removed from the burner. Based on this, the suitable algorithm for pot detection was developed. The microprocessor-based circuit for detection of the pot presence and absence on the burner was designed, built and further implemented. The tests have shown that the circuit worked as it was expected in the laboratory environment, i.e., generated acoustic and light warning signals, switched on the LED and switched off the gas valve when the pot was removed from the burning gas [26]. Repeated tests for small flames have shown that even the use of two thermocouples does not guarantee reliable detection of taking the pot from the burner. Therefore, in this case, the device generated only LED and acoustic warning signals. For middle and big flames, the device worked without any problems and switched off the gas valve when the pot was removed from the burning gas. The above described solution was patented [27]. 

In future work, the reliability of the proposed solution should be improved, i.e., it should be even more insensitive to interference. To this end, we plan to use a potentiometer in the valve to measure the degree of valve opening. The use of IR diodes can allow building more compact construction of the device. However, in this case the problem is where to mount these four IR LEDs in the oven and how to lead the wires to them. An obstacle in using this solution is also the possibility of soiling these diodes and the difficulty of cleaning them. 

## Figures and Tables

**Table 1:** Laboratory investigation results obtained with the use of the IR photodiode.

Distance	Flame	Recorded Signals	Enlarged Fragment of the Course
50 mm	Big	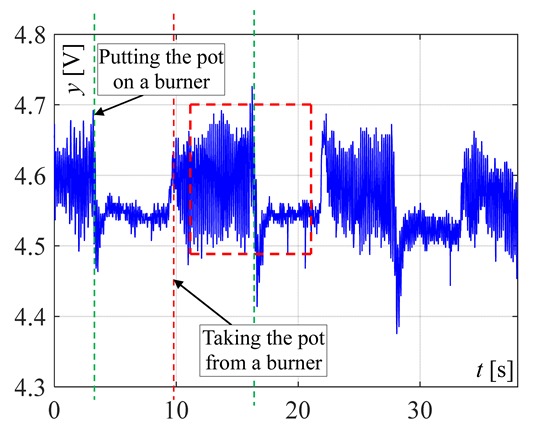	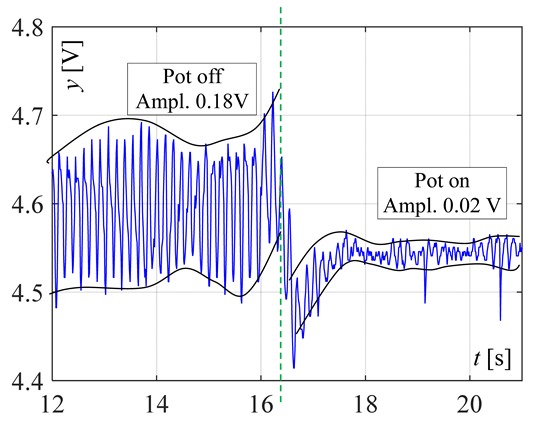
50	Small	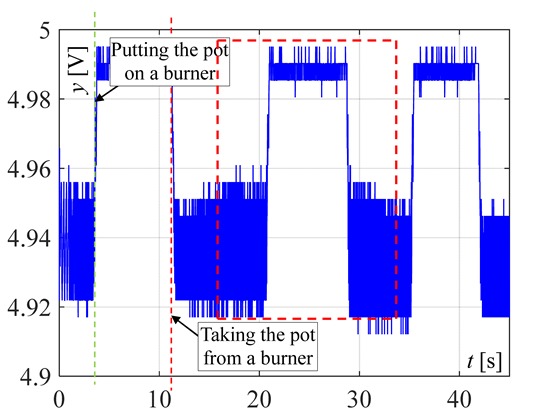	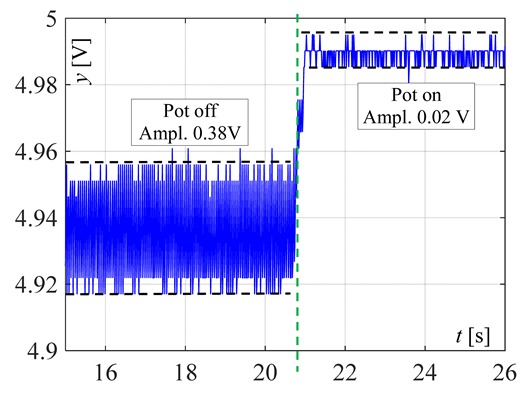
80	Big	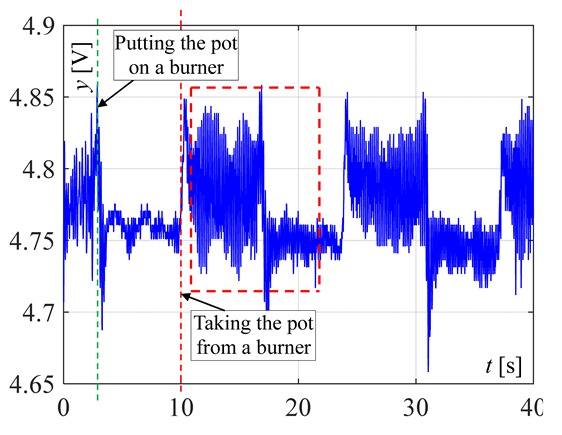	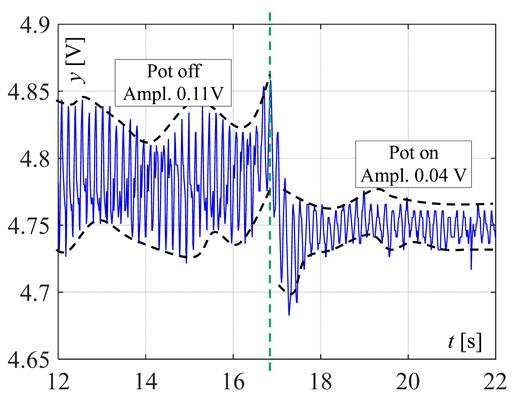
80	Small	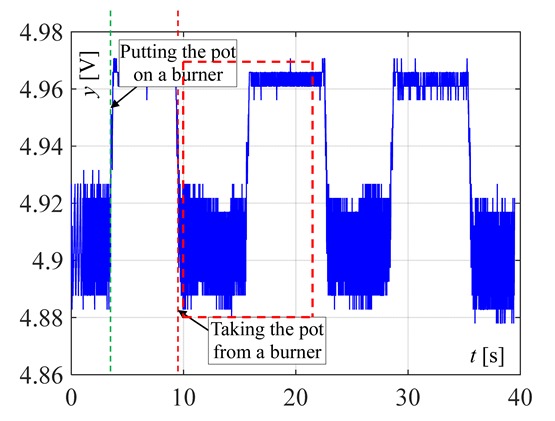	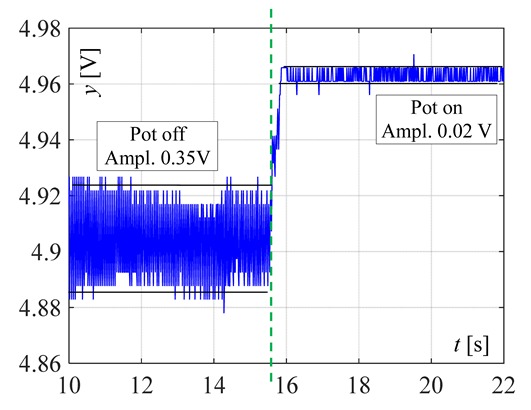
110	Big	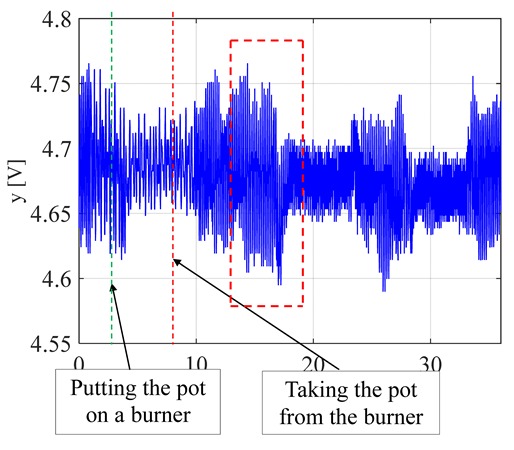	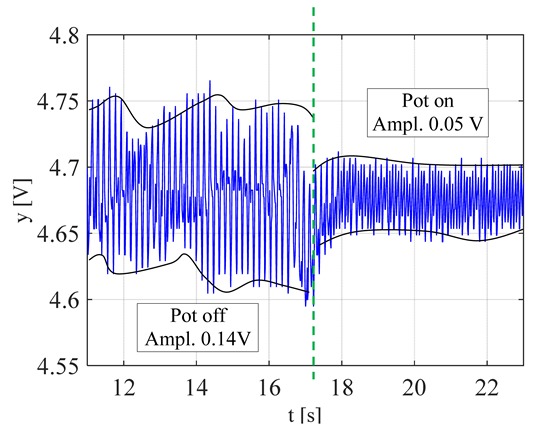
110	Small	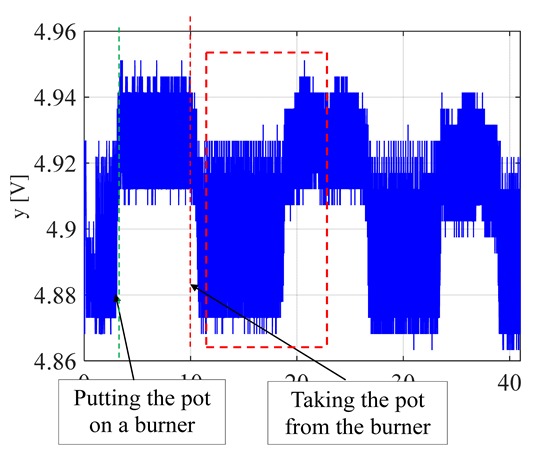	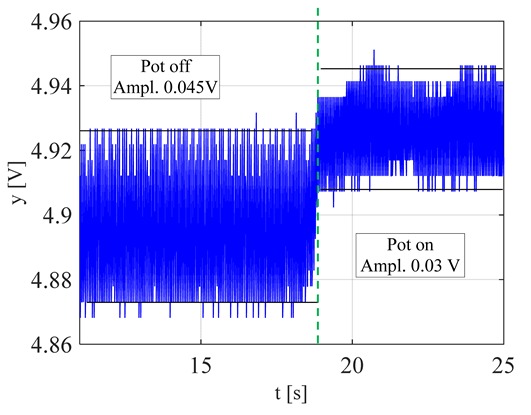

**Table 2:** Amplitudes differences of the recorded signals.

Distance	Flame	Difference
50 mm	Big	0.16 V
50 mm	Small	0.018 V
80 mm	Big	0.07 V
80 mm	Small	0.015 V
110 mm	Big	0.09 V
110 mm	Small	0.015 V
